# Evaluation of Sustained BMP-2 Release Profiles Using a Novel Fluorescence-Based Retention Assay

**DOI:** 10.1371/journal.pone.0123402

**Published:** 2015-04-22

**Authors:** Wonmo Kang, Dong-Sung Lee, Jun-Hyeog Jang

**Affiliations:** 1 Department of Biochemistry, Inha University School of Medicine, Incheon, Republic of Korea; 2 Department of Biomedical Chemistry, Konkuk University, Chung-Ju, Republic of Korea; China Medical University, TAIWAN

## Abstract

The purpose of this study was to develop and characterize a novel fluorescence-based retention assay for the evaluation of the release profile of bone morphogenetic protein-2 (BMP-2) released from bone graft carrier. In this study, we evaluated the binding, release kinetics, and delivery efficacies of BMP-2 incorporated into hydroxyapatite (HA) bone grafts. The evaluation of the release profile of BMP-2 from HA bone grafts using a fluorescence-based retention assay revealed initial burst releases from the HA bone grafts followed by long sustained releases up to 14 weeks. The sustained biological activity of the released BMP-2 from HA bone grafts over the full 14-week period supports a long sustained mechanism via fluorescence-based retention assay. Thus, the results from this study show that BMP-2 could be incorporated into HA bone grafts for sustained release over a prolonged period of time with retention of bioactivity and our fluorescence-based retention assay, which is principally detecting the retention profile of BMP-2 in HA bone grafts, is more accurate than conventionally collecting the released BMP-2 for evaluation of BMP-2 release profiles.

## Introduction

Bone morphogenetic protein-2 (BMP-2) has become the most powerful osteoinductive growth factor for bone regeneration [1]. Currently, recombinant BMP-2 in combination with a collagen sponge has been approved for the treatment of open long bone fractures and combined with a metal cage for spinal fusions [2, 3]. However, due to a short half-life, supraphysiological doses are applied resulting in negative side effects such as ectopic bone formation, or even loss of bone [4, 5]. Current applications include rhBMP-2 loaded in delivery systems to retain rhBMP-2 at the site of injury for a prolonged time frame with a controlled release enhancing the effect [6, 7]. Thus, sustained release of growth factors is a highly desired property of controlled-release materials [6, 8].

Hydroxyapatite (HA), a calcium phosphate crystal that makes up the principal constituent of bone mineral, is a widely-used osteoconductive biomaterial for bone repair [9]. Recently, HA have shown a promise as a scaffolding biomaterial for synthetic bone grafts, and as a bone-mimetic component within composite degradable biomaterials [10]. Therefore, this study aimed to evaluate the control release of rhBMP-2 loaded HA bone graft. To evaluate and visualize the release profile of rhBMP-2 from HA bone graft, we constructed BMP-2 fused to green fluorescent protein (GFP). Fluorescence-based retention assays could be used to detect large amounts of proteins as well as instantly identify the release profile as fluorescence image. The presence of the growth-factor in the bone graft surface was confirmed by fluorescence microscopy using BMP-2_**GFP**_. After 14 weeks of BMP-2_**GFP**_ release, a trace of green fluorescence was still observed on the surface.

The purpose of this study is to evaluate BMP-2’s release kinetics and delivery efficacy using HA bone graft to assess this delivery system’s suitability for bone tissue engineering. To evaluate BMP-2’s release profile from HA bone graft, we developed a novel method using a fluorescence-based retention assay. Until today, protein release profiles from carrier materials have commonly been measured using enzyme-linked immunosorbent (ELISA) assays [11–13]. ELISA-based release assays measure the quantity of released proteins, whereas fluorescent-based retention assays measure the quantity of retained proteins on the carrier materials. Here, BMP-2_**GFP**_ protein was genetically engineered and used for our fluorescent-based retention assay. Furthermore, the biological activity of BMP-2 released from HA bone graft was performed to validate the release kinetics of BMP-2 from HA bone graft and exclude the possible effect of fusion protein on the release kinetics

## Materials and Methods

### Reagents

Commercial BMP-2 was supplied from GENOSS (GENOSS, Suwon, Korea). To assess the release profile of BMP-2, 30 μg of BMP-2 (GENOSS, Suwon, Korea) was used. As bone graft, OSTEON, which was also supplied from GENOSS (GENOSS, Suwon, Korea), was used. In the present study, 0.5 mg or 20 mg OSTEON was used.

### Construction and expression of BMP-2_GFP_ in *E*. *coli*

To construct the BMP-2_GFP_ protein, the cDNA of GFP (CLONTECH) was amplified by polymerase chain reaction (PCR) using a forward primer, 5′-GGAATTCGTGAGCAAGGGCGAGGAG-3′ and reverse primer, 5′-TGAATTCTACTTGTACAGCTCGTC-3′. PCR was carried out in a 30-μL reaction volume containing 50 mM KCl, 10 mM Tris–HCl (pH 8.3), 1.5 mM MgCl_2_, 100 μg/mL gelatin, 0.2 mM deoxyribo nucleotide triphosphates, 1.25 U Taq polymerase (ELPiS Biotech, Daejeon, Korea), and 50 pmol each of the forward and reverse primers. The thermo cycling parameters used in the PCR were as follows: denaturation for 1 min at 94°C, annealing for 1 min at 55°C, and extension for 2 min at 72°C. After 35 cycles, the amplified cDNA was digested. After digestion, PCR products were in-frame-ligated into the *EcoR*I sites of the pBAD-HisA-BMP-2 vector [14], yielding the construct pBAD-His A-BMP-2/GFP.

### Production and purification of BMP-2_GFP_ plasmids

For the expression of BMP-2_GFP_, TOP10 cells were grown overnight in LB-Amp^**+**^ medium at 37°C. When the cultures reached an A_600_ = 0.6, induction was initiated with 0.02% (w/v) l-arabinose as inducer. After 3 h, bacteria were pelleted by centrifugation, lysed, and sonicated. A soluble extract was prepared by centrifugation for 30 min at 6,000 rpm in a refrigerated centrifuge, and the supernatant was transferred to a fresh tube. The crude protein from the sonicated bacterial supernatant was purified through binding of the His_6_ tag (located at the amino-terminal end of the protein) to the nickel-nitrilotriacetic acid resin column, according to the manufacturer’s protocol (Invitrogen, Carlsbad, CA, USA).

### Binding activity by protein concentration

To investigate the maximum binding concentration of BMP-2_GFP_ on bone graft by BMP-2_GFP_ concentration, 0.5 mg bone graft were placed in 24-well plates and incubated with various concentrations of BMP-2_GFP_ (0–35 μg) in stimulated body fluid (SBF) at 37°C. The fluorescent image of BMP-2_GFP_ adsorption to the granules was captured by fluorescence microscopy (Multi-fluorescence, SPOT Advanced, ZEISS, Oberkochen, Germany) and quantified. Fluorescence was excited using a 488 nm laser output and emission was detected using a 510/20 nm bandpass filter.

### Binding activity by temperature

To investigate the optimal binding time of BMP-2_GFP_ on bone graft by temperature, 0.5 mg bone graft were placed in 24-well plates and incubated with 30 μg of BMP-2_GFP_ in SBF at 4°C, 20°C, and 37°C. The fluorescence image of BMP-2_GFP_ adsorption to the granules was captured by fluorescence microscopy and quantified with Quantity One software (Quantity One 1-D analysis software, Bio-Rad).

### *In vitro* release kinetics of BMP-2_GFP_ using a fluorescence-based retention assay

To assess the sustained release profile of BMP-2, 20 mg bone graft were placed in 24-well plates and incubated with 30 μg of BMP-2_GFP_ in SBF at 20°C for 1 day. Then, the sustained release profile of BMP-2 from the bone graft was measured by fluorescence microscopy for 14 weeks. The fluorescence images were captured and the intensities were quantified.

### In vitro release kinetics of BMP-2 and BMP-2_GFP_ using a sandwich ELISA

In a parallel experiment, the ELISA-based release profile of BMP-2 from HA bone grafts was measured using sandwich ELISA (Human BMP-2 ELISA development Kit, PeproTech) for 14 weeks. The *in vitro* release of BMP-2 from HA bone grafts was determined in phosphate-buffered saline (PBS). Each sample was immersed in 100 μL PBS and incubated at room temperature (RT). In addition, every day for 14 weeks, the supernatant of each specimen was collected for sandwich ELISA. Briefly, 100 μL of solution containing the capture antibody (1 μg/mL) was added to 96-well plates and incubated overnight at RT. Then, the wells were washed and blocked with blocking buffer. Next, 100 μL of solution containing released BMP-2_GFP_ was added and incubated at RT for 2 h. After washing, 100 μL of solution containing the detection antibody (1 μg/mL) was added and incubated at RT for 2 h. After washing, the plates were incubated with 100 μL of a solution containing avidin peroxidase (dilution at 1:1500) at RT for 30 min. Following multiple washings, 100 μL of TMB substrate solution (1-Step Ultra TMB-ELISA, PIERCE) was added and incubated at RT for 5 min or until the desired color developed. To stop the reaction, 100 μL of 2 M sulfuric acid was added. Color development was monitored with an ELISA plate reader (Emax, Molecular Devices) at 450 nm.

### Cell culture

C2C12 is a mouse myoblast cell line that is widely used to study the differentiation of myoblasts and osteoblasts, to express various proteins, and to explore mechanistic pathways [19] because these cells have differentiation capability. C2C12 cells were cultured in Dulbecco’s modified eagle medium (DMEM, Welgene, Daegu, Korea) containing 10% (v/v) heat-inactivated fetal bovine serum (Welgene, Daegu, Korea), 100 U/mL penicillin G sodium, 100 μg/mL streptomycin sulfate, and 0.25 μg/mL amphotericin B (Anti-biotic Anti-mycotic Solution, Welgene, Daegu, Korea). C2C12 cells were incubated at 37°C in a humidified atmosphere of 5% CO_2_. When the cells in a culture dish reached confluence, they were detached with trypsin/ethylenediaminetetraacetic acid (EDTA). To induce differentiation of C2C12 cells, low serum medium was used (1% fetal bovine serum).

### Alkaline phosphatase (ALP) assay

ALP is an enzyme reflecting bone induction. To analyze the osteogenic differentiation of C2C12 cells, intracellular ALP activity was determined by the *p*-nitrophenyl-phosphate (pNPP) hydrolysis method using the alkaline phosphate assay kit (Sigma Aldrich, USA).

The experiment was carried out under sterile conditions. ALP activity was measured in 3 different conditions. Firstly, ALP activity was measured in C2C12 cells at various concentrations of BMP-2. C2C12 cells were seeded (1 × 10^4^ cells/well) in 24-well flat-bottomed plates (Nunc, EU) with various amounts of BMP-2 (0, 7.812, 15.625, 31.25, 62.5, 125, 250, 500, and 1000 ng/mL) in differentiation-inducing media for 7 days. Secondly, ALP activity of BMP-2-incorporated bone grafts in C2C12 cells was also measured after incubation with the graft for 1 day. Numerous studies have been reported that most proteins were released from biomaterial within 1 week (early burst mechanism). Thus, we finally investigated the osteogenic differentiation effect of the remaining BMP-2 on the bone graft after releasing for 1 week. Initially, 20 mg bone graft was incorporated with 300 μg/mL of BMP-2_GFP_ in SBF for 1 day and was then allowed to release for 1 week (early pre-released condition). After 1 week, C2C12 cells were seeded at a density of 1 × 10^4^ cells per well with BMP-2-incorporated bone graft in new 24-well plates and incubated in differentiation-inducing media for 7 days. Similarly, the effect of remaining BMP-2 on the bone graft after releasing was measured every week for 14 weeks.

At 7 days, C2C12 cells were washed with PBS and lysed in 1.5 M Tris/HCl (pH 10.2) containing 1 mM ZnCl_2_, 1 mM MgCl_2_ and 1% Triton X-100 at 4°C for 10 min. Following clarification by centrifugation, ALP activity in the cell lysates was measured using an alkaline phosphate assay kit (Sigma Aldrich, USA) according to manufacturer’s instructions. ALP activity was normalized to total protein content of each sample using the Coomassie Plus–The Better Bradford Assay Kit (Thermo Scientific, Illinois, USA).

### Statistics

Experimental results were expressed as the mean ± standard deviation (SD). Statistical analyses were performed using one-way ANOVA (*p<0.05).

## Results

### Construction, expression, and purification of BMP-2_GFP_ protein

**Fig 1:**
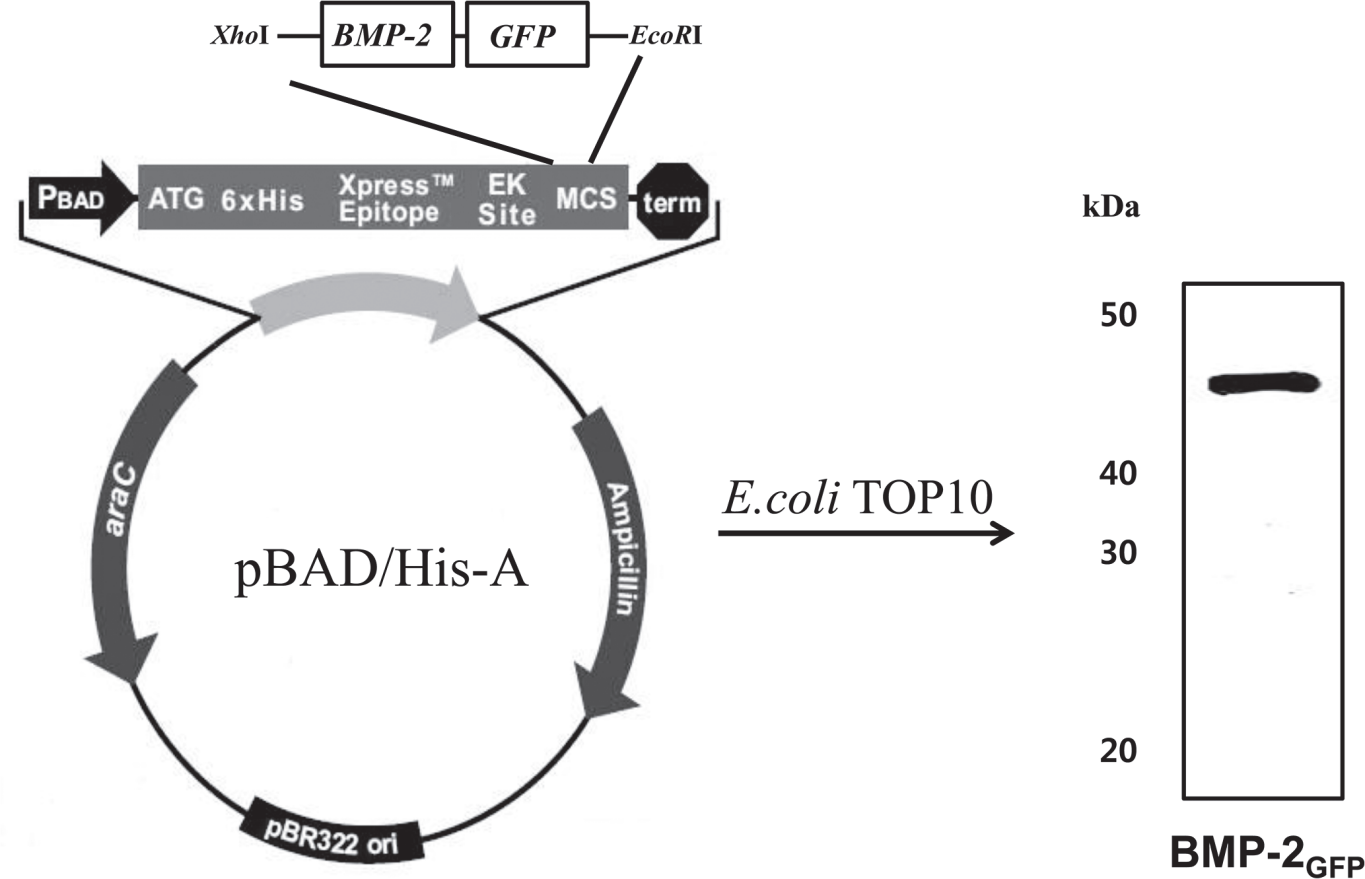
Schematic representation of the BMP-2_GFP_ fusion proteins and Western blotting analysis of BMP-2_GFP_ shown at 45 kDa.

To develop a new method to evaluate the release of BMP-2 from HA bone grafts and to assess the suitability of these HA bone grafts for bone regeneration, we constructed fluorescent BMP-2 fusion protein, BMP-2_GFP_. To maximize protein expression and purification, the fused gene was put under the control of the araBAD promoter for tightly regulated expression and an amino-terminal polyhistidine sequence for affinity purification. Upon induction with L-arabinose, *E*. *coli* TOP 10 produced recombinant proteins. The recombinant BMP-2_GFP_ protein was obtained after affinity purification using a Ni-NTA resin. Protein purity was assessed by SDS-PAGE and estimated to be greater than 95%. The expression of the BMP-2_GFP_ proteins was confirmed by Western blot using a peroxidase conjugate of a monoclonal anti-polyhistidine antibody. The molecular weight of BMP-2_GFP_ was approximately 45 kDa, respectively (Fig 1).

### Binding activity of BMP-2_GFP_ on bone grafts by protein concentrations

**Fig 2:**
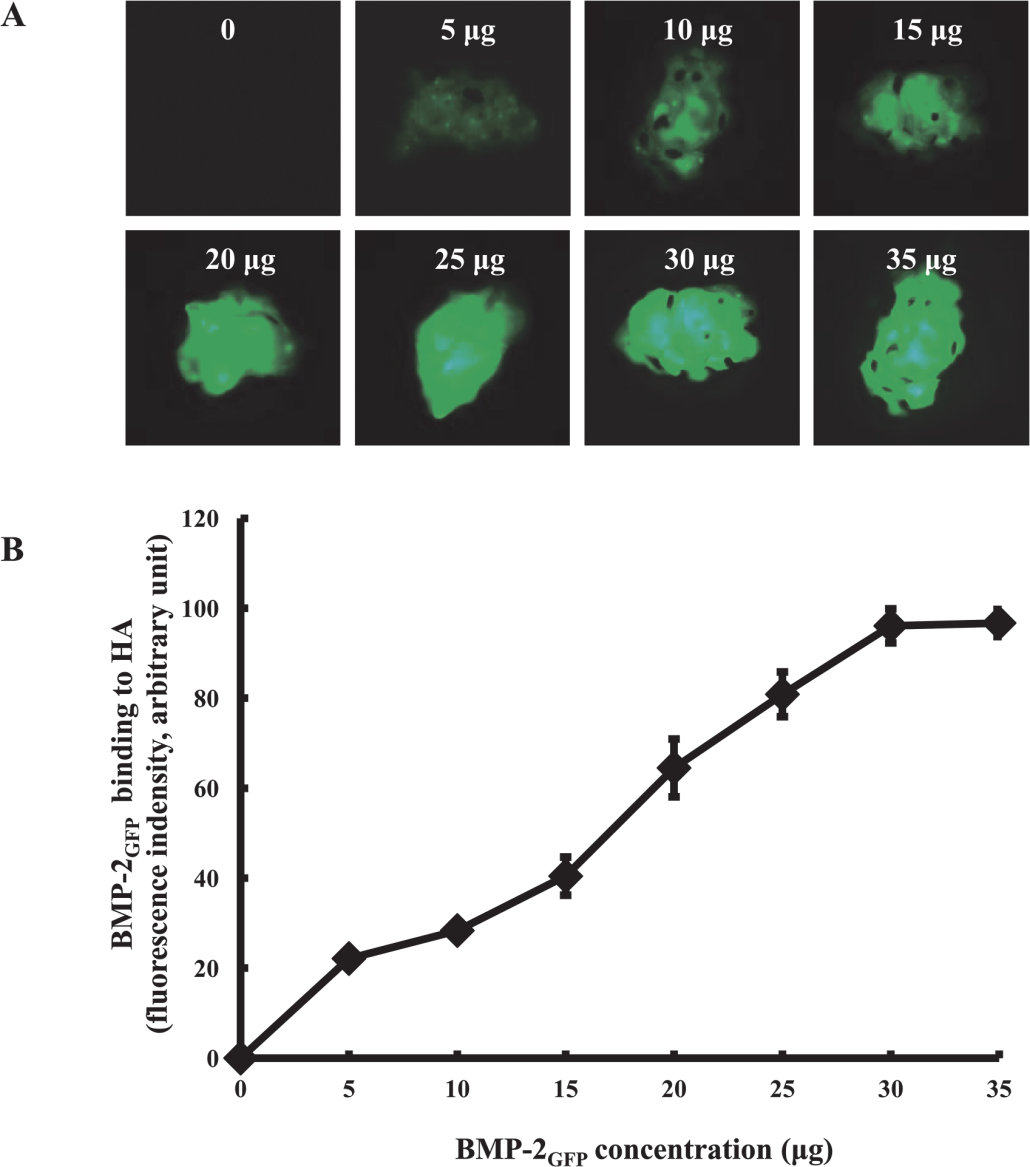
Binding activity of BMP-2_GFP_ on bone graft by protein concentration. A. Representative fluorescence images. B. Fluorescence intensity. HA bone grafts were placed in 24-well plates and adsorbed with a various concentrations of BMP-2_GFP_ (0–35 μg) in SBF at 37°C for 2 h. The fluorescent image of BMP-2_GFP_ adsorption to the granules was captured under fluorescence microscopy and quantified. Results represent the mean ± SD (n = 3).

To evaluate the binding capacity of BMP-2_GFP_ on HA bone graft, HA bone grafts were incubated in SBF with various concentrations of BMP-2_GFP._ The fluorescence intensity of BMP-2_GFP_ on bone grafts significantly increased in a dose-dependent manner, and remained constant above 30 μg (Fig 2). Thus, 30 μg of BMP-2_GFP_ was used in subsequent experiments.

### Binding activity of BMP-2_GFP_ on HA bone grafts by temperature

**Fig 3:**
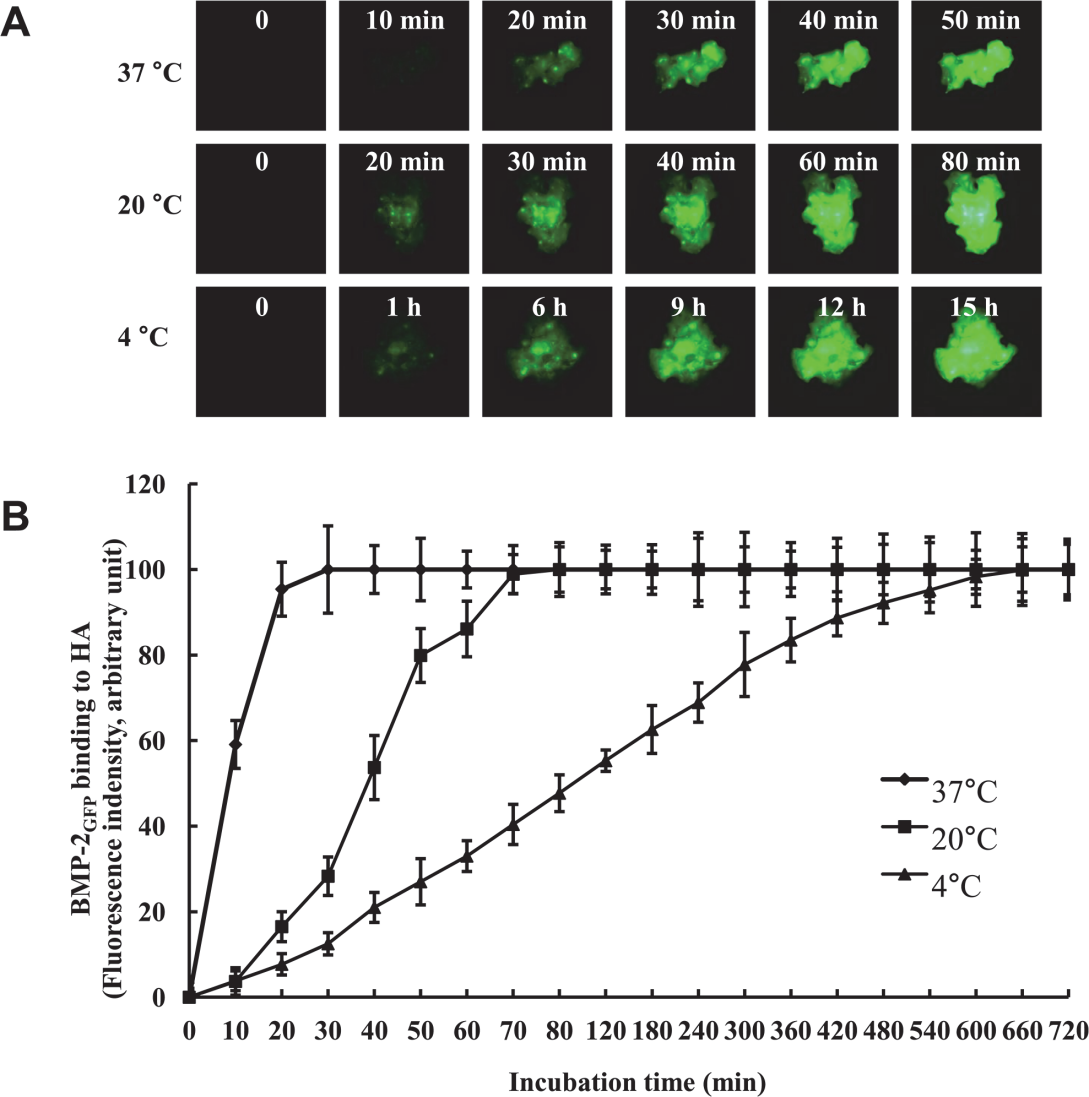
Binding activity of BMP-2_GFP_ on bone graft by temperature. A. Representative fluorescence images. B. Fluorescence intensity. HA bone grafts were placed in 24-well plates and adsorbed with 30 μg of BMP-2_GFP_ in SBF at 4°C, 20°C, and 37°C. The fluorescent image of BMP-2_GFP_ adsorption to the granules was captured under fluorescence microscopy and also quantified. Results represent the mean ± SD (n = 3).

To identify the optimal binding time of BMP-2_GFP_ on HA bone grafs, the HA bone grafts were incubated in SBF with 30 μg of BMP-2_GFP_ at 4°C, 20°C, and 37°C. As shown in Fig 3, >95% of BMP-2_GFP_ was bound to the bone graft within 80 min both at 20°C and 37°C. However, it took over 12 h at 4°C. These results indicate that the binding time of BMP-2_GFP_ on HA bone graft depends on the incubation temperature.

### Release profile of BMP-2 _GFP_ from HA bone graft

The release profiles of BMP-2 from HA bone grafts were fluorescence-based retention assay. In this study, the release profiles of BMP-2_GFP_ from HA bone grafts were expressed as the retention profile of BMP-2_GFP_. In the fluorescence-based retention assay, we initially identified the release with captured fluorescence images of the remained BMP-2_GFP_ in HA bone grafts and confirmed it by quantifying fluorescent intensity.

**Fig 4:**
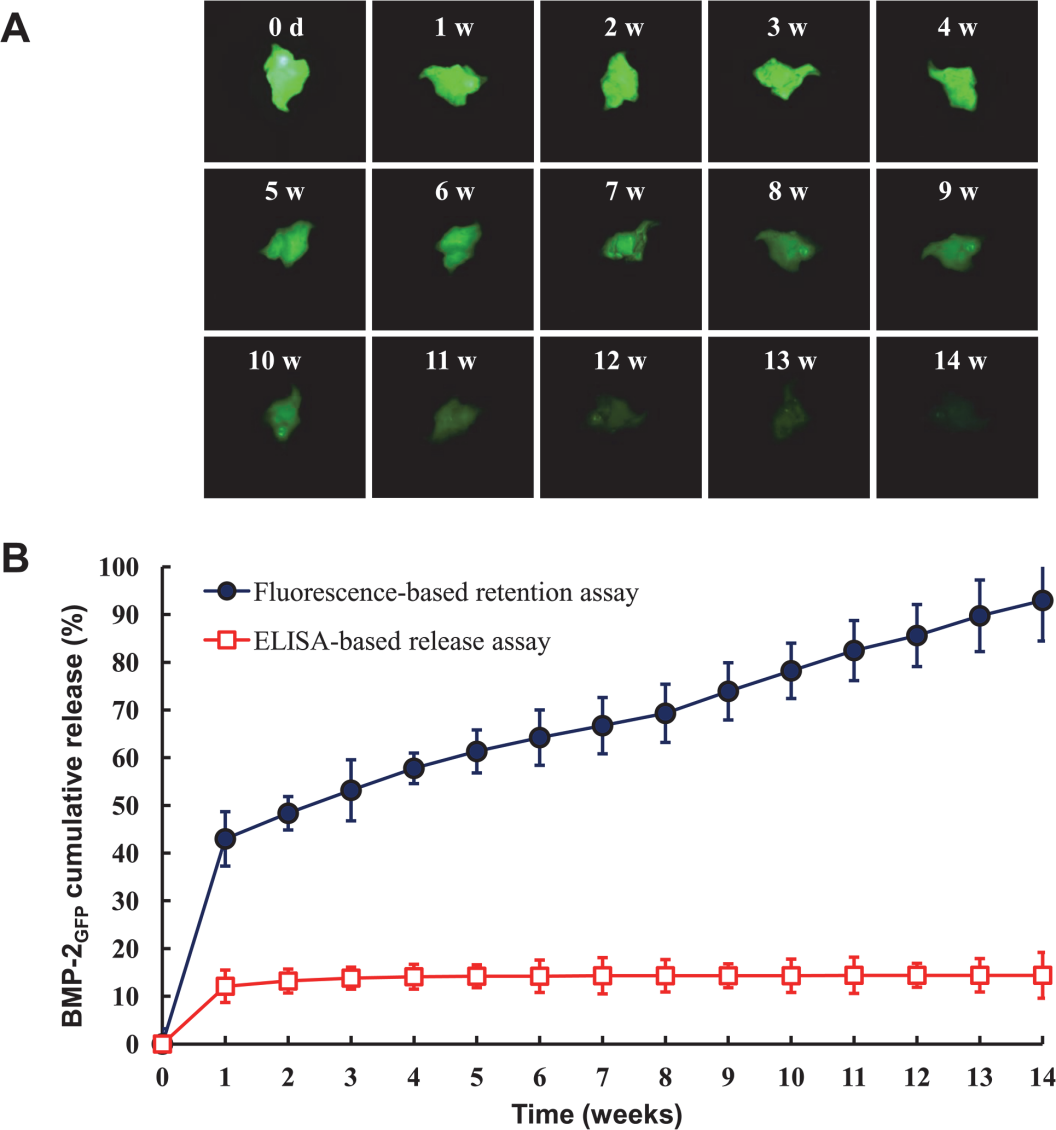
The release profile of BMP-2_GFP_ on bone graft by fluorescence intensity assay. The release profile of BMP-2_GFP_ from bone graft was conversely expressed as the retention profile of BMP-2_GFP_. A. Representative fluorescence images by fluorescence-based retention assay. HA bone grafts were placed in 24-well plates and incubated with 30 μg of BMP-2_GFP_ in SBF at 20°C for 1 day. Then, the sustained release profile of BMP-2_GFP_ from bone grafts was measured by fluorescence microscopy for 14 weeks. The fluorescence image was captured and the fluorescence intensity was normalized with respect to the initial fluorescence. B. Release profile using fluorescence-based retention assay and ELISA. HA bone grafts were placed in 24-well plates and incubated with 30 μg of BMP-2_GFP_ in SBF at 20°C for 1 day. The amount of released BMP-2_GFP_ was quantified via ELISA according to the manufacturer’s instructions. Results represent the mean ± SD (n = 3).

To evaluate the release profile of BMP-2 from HA bone grafts, the release of BMP-2_GFP_ loaded onto HA bone grafts was investigated over 14 weeks. The amount of BMP-2_GFP_ released from HA bone grafts was first determined using an ELISA. ELISA-based release assay showed an early burst release profile of BMP-2_GFP_ (12.1% of the total loaded BMP-2_GFP_) for 1 week. After 1 week, the remaining amount of BMP-2_GFP_ released from HA bone grafts was barely detectable, detecting 14.7% of the total loaded BMP-2_GFP_ in HA bone grafts over 14 weeks (Fig 4). Similar results were obtained using BMP-2 (data not shown).

In contrast to ELISA-based release profile, the fluorescence-based retention assay showed early burst releases (42.9% of the total loaded BMP-2_GFP_) from HA bone grafts for 1 week followed by sustained releases with a controlled release rate of 6.7% per week up to 14 weeks (Fig 4B). In the fluorescence-based retention assay, the amount of released BMP-2_GFP_ from the HA bone grafts was calculated from the retained BMP-2_GFP_ on the HA bone grafts.

Consequently, the fluorescence-based retention assay detected most of the BMP-2_GFP_ and revealed a sustained release profile for 14 weeks *in vitro*. These results indicate that the measurement of retention profile of BMP-2 by using a fluorescence-based retention assay could be an effective technique.

### Biological activity of the released rhBMP-2 from bone grafts

**Fig 5:**
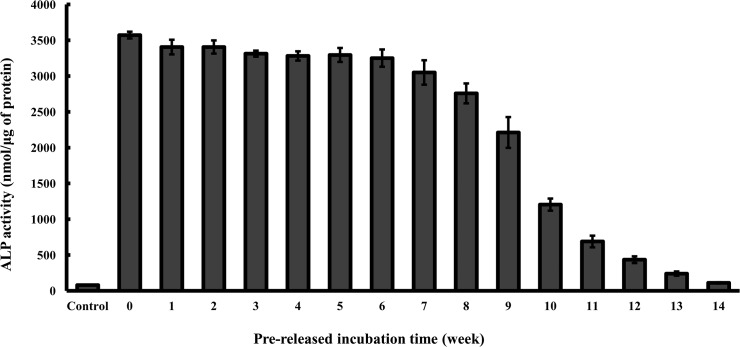
ALP activity assay of pre-released BMP-2 incorporated bone graft. ALP activity was quantified in C2C12 cells exposed to BMP-2 incorporated HA bone grafts after pre-release in SBF for indicated times or HA bone grafts only (control). Results represent the mean ± SD (n = 3).

ALP activity was assessed as an early indicator of the osteoblastic lineage to study the effect of BMP-2 an osteoblast differentiation. To validate the sustained release mechanism of actual rhBMP-2 from HA bone grafts as observed in the fluorescence-based retention assay, the ALP activity of rhBMP-2 after pre-release in SBF over a period of 14 weeks was measured. The released rhBMP-2 retained its biological activity over 14 weeks as indicated by the increased ALP response over basal level of the cells, consistent with sustained release profile observed in the fluorescence-based retention assay (Fig 5). Interestingly, the ALP increase by the rhBMP-2 released from HA bone grafts was significantly higher compared to comparable rhBMP-2 concentrations of the dose-response curve that had been directly added to the culture medium of the cells (data not shown). These results of the fluorescence-based retention assay clearly show that biologically active rhBMP-2 can be released from HA bone grafts over a period of about 14 weeks.

## Discussion

The osteo-inductive factor BMP-2 is in clinical use for the treatment of bone fractures [15]. However, one of the main drawbacks to the application of these growth factors is to increase the efficacy of growth factor therapy and to reduce the needed dosage by sustained release from carrier. In fact, growth factors are particularly vulnerable to degradation or inactivation in the cell medium by molecules such as serum proteins [4]. Therefore, the combination of growth factors and biomaterials contributes to the higher biological activity via sustained delivery [16]. Here, we investigated the release profile of BMP-2 from bone grafts through a fluorescence-based retention assay.

Until today, ELISA-based release assays are a common method for evaluating the release of growth factors from biomaterials [11–13]. However, growth factors are known to be sticky proteins. At low concentrations of growth factors, a significant fraction of the growth factors are bound to a variety of surfaces, including polystyrene and glass [17, 18]. Therefore, it is not surprising that the fluorescence-based retention assay detected 93% of the total loaded BMP-2_GFP_ in HA bone grafts over 14 weeks. In contrast, the ELISA-based release assay detected 14.7% of the total loaded BMP-2_GFP_ in HA bone grafts over 14 weeks. Draenert et al. showed similar release profile of BMP-2 from HA bone grafts with a release of several days in ELISA-based release assay compared to the ELISA results of our study [19].

Matsumoto et al. studied the release kinetics of a standard protein from HA using cytochrome c and showed 80% release of the loaded protein from HA [20]. Hänseler et al. also showed 100% release of loaded ^125^I-BMP-2 from apatite bone grafts [21]. This result correlates with our 93% release data of total loaded BMP-2_GFP_ from HA bone grafts. Therefore, it is unlikely that BMP-2_GFP_ photobleaches over 14 weeks without release from HA bone grafts. These results suggest that the fluorescence-based retention assay, which is principally detecting the retention profile of BMP-2_GFP_ in HA bone grafts, could be more effective than conventionally collecting the released BMP-2 for evaluation of BMP-2 release profiles because of its stickiness.

Together with the release profile, the ALP activity of BMP-2 was measured in C2C12 cells. Initially, ALP activity at various concentrations of BMP-2 was measured in C2C12 cells. BMP-2 dose-dependently induced osteogenic differentiation of C2C12 cells. Secondly, BMP-2-incorporated HA bone grafts increased the ALP and mineralization activity in C2C12 cells as compared to the controls. Most importantly, ALP activities of remaining BMP-2 were measured in C2C12 cells to validate the sustained biological activity in the fluorescence-based retention assay. Surprisingly, the remaining BMP-2 also showed higher ALP activity than that of the controls. Although the ALP activity of remaining BMP-2 decreased time-dependently decreased in C2C12 cells, it was still higher than that of the control (Fig 5).

In this study, our fluorescence-based retention assay, which is principally detecting the remained BMP-2, is accurate for BMP-2 release profile. In addition, the fluorescence-based retention assay could detect a relatively large amount as well as instantly identify the release profile as fluorescence image.

## References

[1] Bessho K, Konishi Y, Kaihara S, Fujimura K, Okubo Y, Iizuka T. Bone induction by Escherichia coli-derived recombinant human bone morphogenetic protein-2 compared with Chinese hamster ovary cell-derived recombinant human bone morphogenetic protein-2. Br J Oral Maxillofac Surg. 2000; 38: 645–649. 11092786 10.1054/bjom.2000.0533

[2] Carragee EJ, Hurwitz EL, Weiner BK. A critical review of recombinant human bone morphogenetic protein-2 trials in spinal surgery: emerging safety concerns and lessons learned. Spine J. 1016; 11: 471–491. 10.1016/j.spinee.2011.04.023 21729796

[3] Carragee EJ, Hurwitz EL, Weiner BK. A critical review of recombinant human bone morphogenetic protein-2 trials in spinal surgery: emerging safety concerns and lessons learned. Spine J. 2011; 11: 471–491. 10.1016/j.spinee.2011.04.023 21729796

[4] Brekke JH, Toth JM. Principles of tissue engineering applied to programmable osteogenesis. J Biomed Mater Res. 1998; 43: 380–398. 9855197 10.1002/(sici)1097-4636(199824)43:4<380::aid-jbm6>3.0.co;2-d

[5] Fu YC, Nie H, Ho ML, Wang CK, Wang CH. Optimized bone regeneration based on sustained release from three-dimensional fibrous PLGA/HAp composite scaffolds loaded with BMP-2. Biotechnol Bioeng. 2008; 99: 996–1006. 17879301 10.1002/bit.21648

[6] Seeherman H, Wozney JM. Delivery of bone morphogenetic proteins for orthopedic tissue regeneration. Cytokine Growth Factor Rev. 2005; 16: 329–345. 15936978 10.1016/j.cytogfr.2005.05.001

[7] Lieberman JR, Daluiski A, Einhorn TA. The role of growth factors in the repair of bone. Biology and clinical applications. J Bone Joint Surg Am. 2002; 1032–1044. 10.2106/00004623-200206000-00022 12063342

[8] Garcia P, Pieruschka A, Klein M, Tami A, Histing T, Holstein JH, et al Temporal and spatial vascularization patterns of unions and nonunions: role of vascular endothelial growth factor and bone morphogenetic proteins. J. Bone Joint Surg. Am. 2012; 94: 49–58. 10.2106/JBJS.L.00240 22218382

[9] Tampieri A, Celotti G, Landi E. From biomimetic apatites to biologically inspired composites. Anal Bioanal Chem. 2005; 381: 568–576. 15696277 10.1007/s00216-004-2943-0

[10] Hutmacher DW, Schantz JT, Lam CX, Tan KC, Lim TC. State of the art and future directions of scaffold-based bone engineering from a biomaterials perspective. J Tissue Eng Regen Med. 2007; 1: 245–260. 18038415 10.1002/term.24

[11] Du P, Hwang MP, Noh YK, Subbiah R, Kim IG, Bae SE, et al Fibroblast-derived matrix (FDM) as a novel vascular endothelial growth factor delivery platform. J Control Release. 2014; 2014: 122–129. 10.1016/j.jconrel.2014.08.026 25194780

[12] Knaack S, Lode A, Hoyer B, Rosen-Wolff A, Gabrielyan A, Roeder I, et al Heparin modification of a biomimetic bone matrix for controlled release of VEGF. J Biomed Mater Res A. 2014; 102: 3500–3511. 10.1002/jbm.a.35020 24178515

[13] Poldervaart MT, Gremmels H, van Deventer K, Fledderus JO, Oner FC, Verhaar MC, et al Prolonged presence of VEGF promotes vascularization in 3D bioprinted scaffolds with defined architecture. J Control Release. 2014; 184: 58–66. 10.1016/j.jconrel.2014.04.007 24727077

[14] Kim JE, Lee EJ, Kim HE, Koh YH, Jang JH. The impact of immobilization of BMP-2 on PDO membrane for bone regeneration. J Biomed Mater Res A. 2012; 100: 1488–1493. 10.1002/jbm.a.34089 22396132

[15] Jones AL, Bucholz RW, Bosse MJ, Mirza SK, Lyon TR, Webb LX, et al Recombinant human BMP-2 and allograft compared with autogenous bone graft for reconstruction of diaphyseal tibial fractures with cortical defects. A randomized, controlled trial. J Bone Joint Surg Am. 2006; 88: 1431–1441. 16818967 10.2106/JBJS.E.00381

[16] Jeon O, Song SJ, Yang HS, Bhang SH, Kang SW, Sung MA, et al Long-term delivery enhances in vivo osteogenic efficacy of bone morphogenetic protein-2 compared to short-term delivery. Biochem Biophys Res Commun. 2008; 369: 774–780. 10.1016/j.bbrc.2008.02.099 18313401

[17] Smith JC, Singh JP, Lillquist JS, Goon DS, Stiles CD. Growth factors adherent to cell substrate are mitogenically active in situ. Nature. 1982; 296: 154–156. 6278315 10.1038/296154a0

[18] Bothwell MA, Shooter EM. Dissociation equilibrium constant of beta nerve growth factor. J Biol Chem. 1977; 252: 8532–8536. 925010

[19] Draenert FG, Nonnenmacher AL, Kammerer PW, Goldschmitt J, Wagner W. BMP-2 and bFGF release and in vitro effect on human osteoblasts after adsorption to bone grafts and biomaterials. Clin Oral Implants Res. 2012; 24: 750–757. 10.1111/j.1600-0501.2012.02481.x 22524399

[20] Matsumoto T, Okazaki M, Inoue M, Yamaguchi S, Kusunose T, Toyonaga T, et al Hydroxyapatite particles as a controlled release carrier of protein. Biomaterials. 2004; 25: 3807–3812. 15020156 10.1016/j.biomaterials.2003.10.081

[21] Hanseler P, Ehrbar M, Kruse A, Fischer E, Schibli R, Ghayor C, et al Delivery of BMP-2 by two clinically available apatite materials: In vitro and in vivo comparison. J Biomed Mater Res A. 2014; 2014: 35211. 10.1002/jbm.a.35211 24771467

